# Investigation of Non-invasive Continuous Body Temperature Measurements in a Clinical Setting Using an Adhesive Axillary Thermometer (SteadyTemp®)

**DOI:** 10.3389/fdgth.2021.794274

**Published:** 2021-12-14

**Authors:** Johannes Boyer, Jakob Eckmann, Karl Strohmayer, Werner Koele, Moritz Federspiel, Michael Schenk, Gregor Weiss, Robert Krause

**Affiliations:** ^1^Department of Infectious Diseases and Tropical Medicine, Medical University of Graz, Graz, Austria; ^2^SteadySense GmbH, Seiersberg-Pirka, Austria; ^3^Das Kinderwunsch Institut Schenk GmbH, Dobl, Austria; ^4^BioTechMed Graz, Graz, Austria

**Keywords:** temperature, measurements, continuous, wearable devices, fever, axillary

## Abstract

Since the human body reacts to a variety of different diseases with elevated body temperature, measurement of body temperature remains relevant in clinical practice. The absolute temperature value for fever definition is still arbitrary and depends on the measuring site, as well as underlying disease and individual factors. Hence, a simple threshold for fever definition is outdated and a definition which relies on the relative changes in the individual seems reasonable as it takes these individual factors into account. In this prospective multicentric study we validate an adhesive axillary thermometer (SteadyTemp®) which allows continuous non-invasive temperature measurements. It consists of a patch to measure temperature and a smartphone application to process and visualize gathered data. This article provides information of the new diagnostic possibilities when using this wearable device and where it could be beneficial. Furthermore, it discusses how to interpret the generated data and when it is not practical to use, based on its characteristics and physiological phenomena.

## Introduction

Body temperature measurements are used to determine the health status of humans. Since the body reacts to a variety of different diseases with elevated body temperature, measurement of body temperature remains relevant in clinical practice. Normal body temperature is considered as 37.0°C (98.6°F) (1). Fever is often defined as an elevated body temperature ≥38°C (100.4°F) (1) due to an increase of the central thermoregulation set point (2). Detection of these elevated body temperature values almost certainly triggers further diagnostics and therapeutic measures, dependent on the prevailing health status. Therefore, the evaluation of the body temperature of outpatients and hospitalized individuals is still common and useful practice in the clinical routine.

The absolute temperature value for fever definition is still arbitrary and depends on the measuring site, as well as underlying disease and individual factors (1, 3). Studies have shown that normal temperature values vary widely between individuals (4, 5) and therefore, a simple threshold for fever definition seems outdated (6) while a patient-specific definition of fever seems to be more accurate.

Temperature measurements of hospitalized patients are mainly carried out by nurses and are time consuming by using contact or infrared thermometers. Due to the extensive nursing workload, it is important to optimize daily work to ensure high quality in patient care. In addition, up to date temperature measurement systems used on normal wards or outpatients settings provide single temperature measurements on certain timepoints whereas continuous and more accurate temperature profiles are obtained on intensive care units (ICU).

Wearable devices to continuously monitor the vital signs of an individual (e.g., blood pressure, heart rate, skin temperature) are a field of research of the last decade. The recent data suggests that wearable devices can improve clinical outcomes by reacting quicker to changes in vital signs. However, high-quality studies in terms of clinical outcomes are still lacking (7–9).

In this study, we validated the SteadyTemp®-system, a medical device for measuring continuous axillary temperature. It consists of a patch to measure temperature and a smartphone application to process and visualize gathered data. It was hypothesized, that the SteadyTemp®-system generates comparable results to established methods of temperature measurement. The primary outcome was agreement of SteadyTemp®-measurement with conventional axillary or bladder temperature measurement systems. Secondary outcomes were comfort and side effects during measurements.

## Materials and Methods

The study was a prospective multicenter study at the Intensive Care Unit (ICU), Department of Internal Medicine at the Medical University of Graz for inclusion of febrile patients and “Das Kinderwunsch Institut Schenk GmbH” for the non-febrile subjects. The study was approved by the ethical committee of the Medical University of Graz, Austria (approval number: 32-503 ex 19/20).

The primary endpoint was agreement of body temperature provided by SteadyTemp®-system and a conventional axillary thermometer (additionally with bladder catheter measurement values in the febrile patients) using a Bland-Altman plot.

### Study Population

A total of 105 participants were required, with a febrile proportion ranging from 30 to 50% (10). Inclusion and exclusion criteria were slightly different for the febrile and the non-febrile group.

#### Inclusion Criteria and Exclusion Criteria

Inclusion criteria were an age older than 5 years and signed informed consent. Unconscious patients at ICU could be included during their sedation or coma with efforts to obtain the patients agreement after arousal. For the febrile group at the ICU an elevated core temperature measurement of ≥38°C was required. For the non-febrile group an absence of febrile disease within 2 weeks prior to study inclusion was required.

Exclusion criteria were skin diseases or wounds that could affect the outcome of the study, pregnancy or breastfeeding as well as known allergies to adhesive plaster or components of it. Also subjects with medication which could impair or falsify the result of the study were not included. Severe, acute or chronic disease were an exclusion criterion only for the non-febrile group.

### Recruitment

The Recruitment was performed differently for the febrile and the non-febrile group.

#### Febrile Patients

After screening for inclusion and potential exclusion criteria and obtaining the informed consent patients at the ICU were included in the study. Sedated or unconscious patients were included and asked for permission to use their data after their arousal according to the approval of the ethical committee.

#### Non-febrile Subjects

After screening for inclusion and potential exclusion criteria potential participants were asked for study participation at “Das Kinderwunsch Institut Schenk GmbH.”

### Temperature Measurement

The temperature measurement was performed with the adhesive axillary thermometer and was compared with an axillary thermometer (Beurer FT 13 (Beurer GmbH, Ulm, Germany) or the Domotherm Rapid 10S (Uebe Medical GmbH, Külsheim, Germany) or a bladder catheter (Philips IntelliVue Monitoring system plus Medovis Silicone Catheter; Philips, Amsterdam, Netherlands).

#### Adhesive Axillary Thermometer (SteadyTemp®-System, SteadySense GmbH, Graz, Austria)

**Figure 1:**
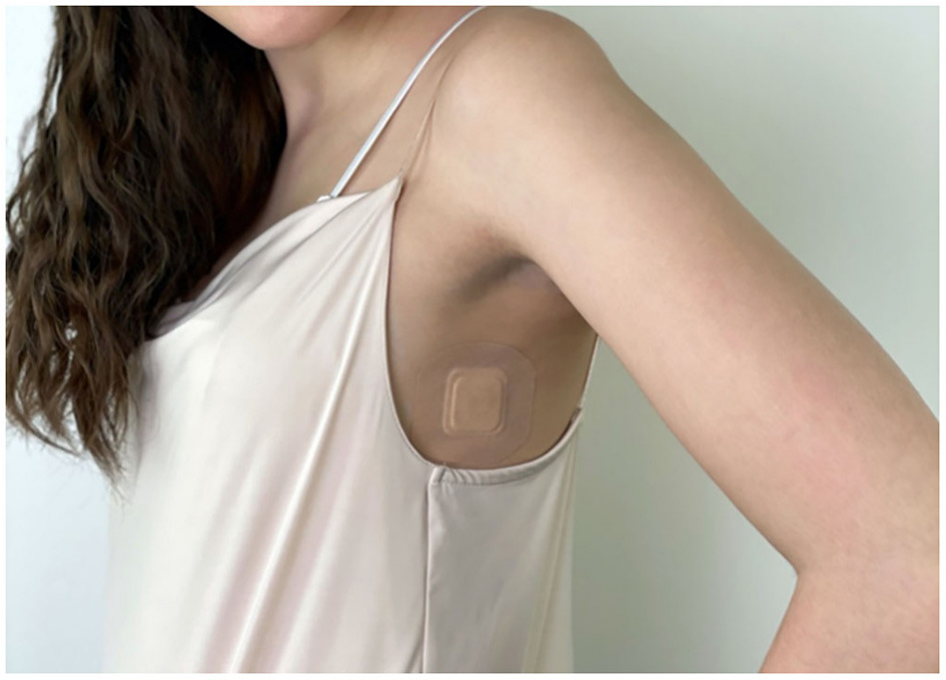
Applied patch in typical location.

**Figure 2:**
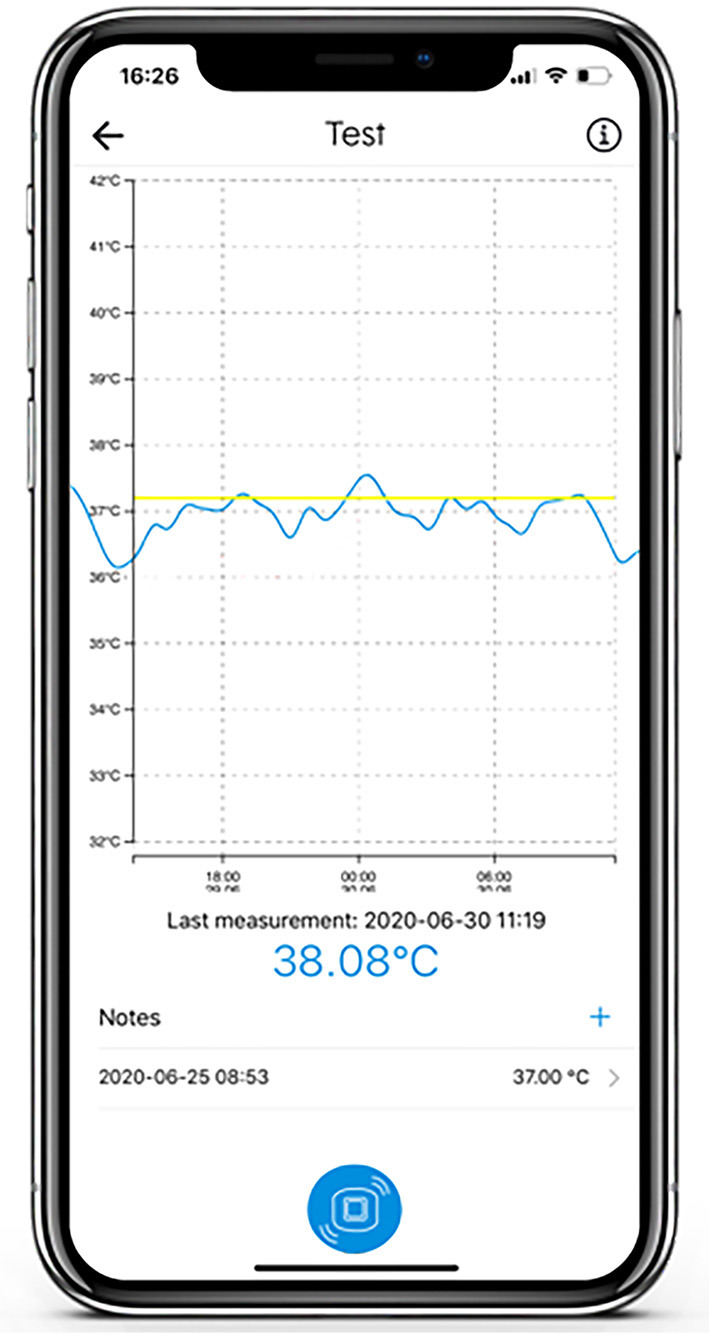
Surface of the Steady Temp app.

The adhesive axillary thermometer is a patch with a precise temperature sensor embedded in adhesive materials, and the associated SteadyTemp® smartphone application, as depicted in Figures 1, 2. The patch is operating as a continuous axillary thermometer in direct mode, while the SteadyTemp® mobile application works as an interface between the patch and the user. After activating the patch via smartphone through near-field-communication (NFC), the patch measures and stores a raw temperature value every 5 min. The raw values are stored in the patch itself and can be gathered via NFC-readout with the corresponding smartphone application. Afterwards, the raw values are translated into temperature values, processed, and visualized by the application as a temperature curve on the smartphone. The adhesive axillary thermometer, i.e., the sensor patch is comprised of certified biocompatible adhesive materials encasing and isolating the sensoric components from ingress of fluids, thus the sensor patch is IPX5 certified.

Within a laboratory setting, sensor patches underwent temperature testing in a waterbath (Biosan WB-4MS). The sensoric components of the sensor patch were submerged in the waterbath, the waterbath temperature was set to the bottom, mid and upper limit of the defined measurement range, i.e., 30, 36, and 42°C, respectively. Additionally, the water surface of the waterbath was partially exposed to an environmental temperature of 15°C using a climatic chamber (CTS T-40/200/S) to simulate the influence of a temperature difference on the sensor patch under quasi-realistic conditions.

Within this test setting, all patches remained within 0.2°C from the reference temperature source at all investigated temperature levels upon achieving thermal stability of the waterbath.

Furthermore, the time response of the sensor patches was investigated during this test setting. The sensor patches showed immediate response to an increase of the reference temperature source. It shall be mentioned that the time response of the patches depends on the selection of the measurement interval. For this test, the measurement interval was set to 60 s. In the normal use case, the measurement interval is defined with 300 s.

According to the device's (SteadyTemp®) intended use, the maximum application duration is defined with 7 days. The limitation is a tradeoff between the continuous measurement and the permanent adhesion of the patch on the wearer's skin.

#### Reference Methods

The SteadyTemp®-system has been compared to an established method of axillary temperature measurement using the Beurer FT 13 (Beurer GmbH, Ulm, Germany) or the Domotherm Rapid 10S (Uebe Medical GmbH, Külsheim, Germany) electronic axillary thermometers, both with an accuracy of ±0.1°C for the range of 35.5–42 and ±0.2°C for other temperature ranges according to the manufacturer. In febrile patients, additional invasive temperature monitoring via bladder catheter measurement (Philips IntelliVue Monitoring system plus Medovis Silicone Catheter; Philips, Amsterdam, Netherlands) was used with an accuracy of ±0.1°C (±0.2°C) according to the manufacturer. Bladder catheter measurement is considered as a surrogate for pulmonary artery catheter measurement (1, 11–13), which is the gold standard for determination of core temperature (1, 11).

### Procedure of Temperature Measurements

Temperature measurements for the febrile and non-febrile group were executed differently due to study design. Per subject, at least four measurements with the axillary reference thermometer were taken. The gathered data were then timely matched. The different procedures are defined in this section.

#### Febrile Patients

The participants at the ICU were in a stable indoor environment with room temperature of 22.8–23.1°C. The patch was placed centrally in the middle axillary line, approximately 3–5 cm underneath the axilla. After activating the patch using the smartphone application, comparative axillary measurements were performed twice per day using the FT 13 Beurer thermometer. During axillary measurements, the thermometer was placed in the armpit at the patch's side with the sensor near the axillary artery, covered by skin for 5 min. In all except one febrile patient, a bladder catheter was utilized and core temperature values were recorded every 30 min. Sedated patients were moved routinely within the bed by nursing staff every 4 h to avoid skin ulcers.

#### Non-febrile Subjects

The participants were brought to an indoor environment with no active climate control and closed windows. In this environment, the patch was applied in the same manner as for the febrile group. After patch application, the participants were instructed to not perform any extensive physical activities during the measurement duration of two hours. Twenty-five minutes after the patch was applied, the participants were instructed to apply the axillary thermometer for a preheating phase of 5 min (Beurer FT 13 thermometer) as specified by the manufacturer, on the same body side as the patch. After the preheating was concluded, a measurement with the axillary thermometer was taken. This procedure was repeated after 55, 85, and 115 min. Thereafter, the patch was detached by a medical professional and the skin was investigated for possible skin irritations.

### Statistics

According to OENORM (10) a minimum of 105 participants, 30–50% febrile, are required to validate temperature systems like the SteadyTemp®-system. Primary endpoint was defined as a clinical bias, within the limits of agreement against the reference methods, using a Bland-Altman plot (14). Bladder catheter measurements were only available for febrile patients. Sensitivity and specificity were calculated using a chi-squared test.

## Results

In the final analysis, 113 subjects, 36 febrile and 77 non-febrile were included. The relatively unspecific criteria resulted in a high heterogeneity of febrile patients with a broad spectrum of primary diagnoses. This includes patients with the leading causes: status post cardiopulmonary resuscitation (CPR) (10 patients), sepsis (6 patients), and acute coronary syndrome (ACS) (5 patients). Other main diagnoses were pneumonia (3 patients), COVID-19 (2 patients), decompensated cirrhosis hepatis (1 patient), thyreotoxicosis (1 patient), anaphylactic shock (1 patient), decompensated chronic kidney disease (1 patient), hyperkalemia (1 patient), pulmonary artery embolism (1 patient), gastrointestinal bleeding (1 patient), hemolytic anemia (1 patient), pancreatitis (1 patient), and myasthenia gravis (1 patient). From these patients 24 (66.67%) received norepinephrine and 19 (52.78%) anti-pyretics. In the febrile group, 3 patients were excluded from the study due to death before generating sufficient data, 1 due to withdrawal after arousal. Table 1 presents a summary of the patient characteristics of the febrile group.

**Table 1:** Characteristics of the febrile group.

**Characteristics of febrile group (*****n*** **=** **36)**	
Sex	9 female (25%)
Age (years)	63.61 (27–85)
BMI (kgm^−2^)	28.22 (16.4–43.6)
Anti-pyretics	19 (52.78%)
Norepinephrine	24 (66.67%)

**Figure 3:**
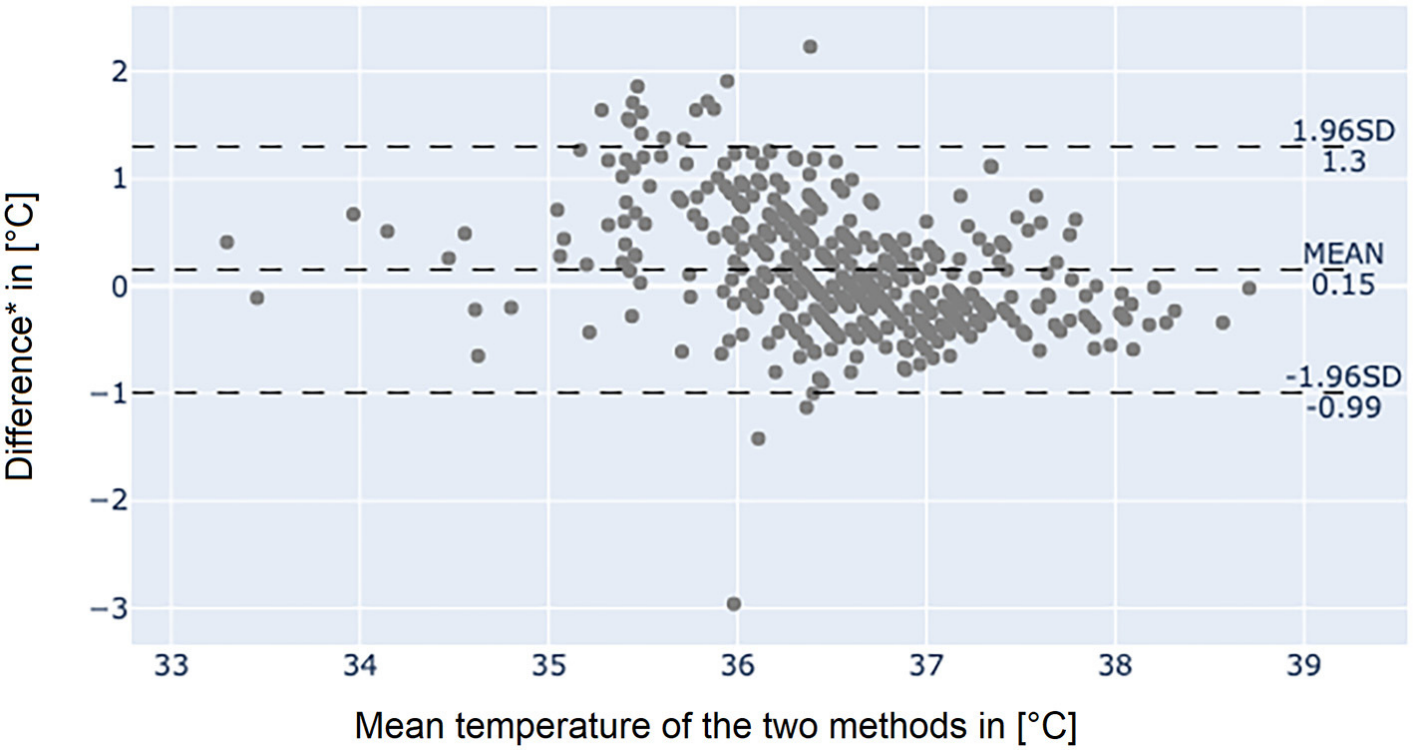
Bland-Altman plot between the conventional axillary method and the adhesive axillary thermometer Steady Temp. *Conventional axillary temperature values minus adhesive axillary thermometer values. SD, standard deviation.

**Figure 4:**
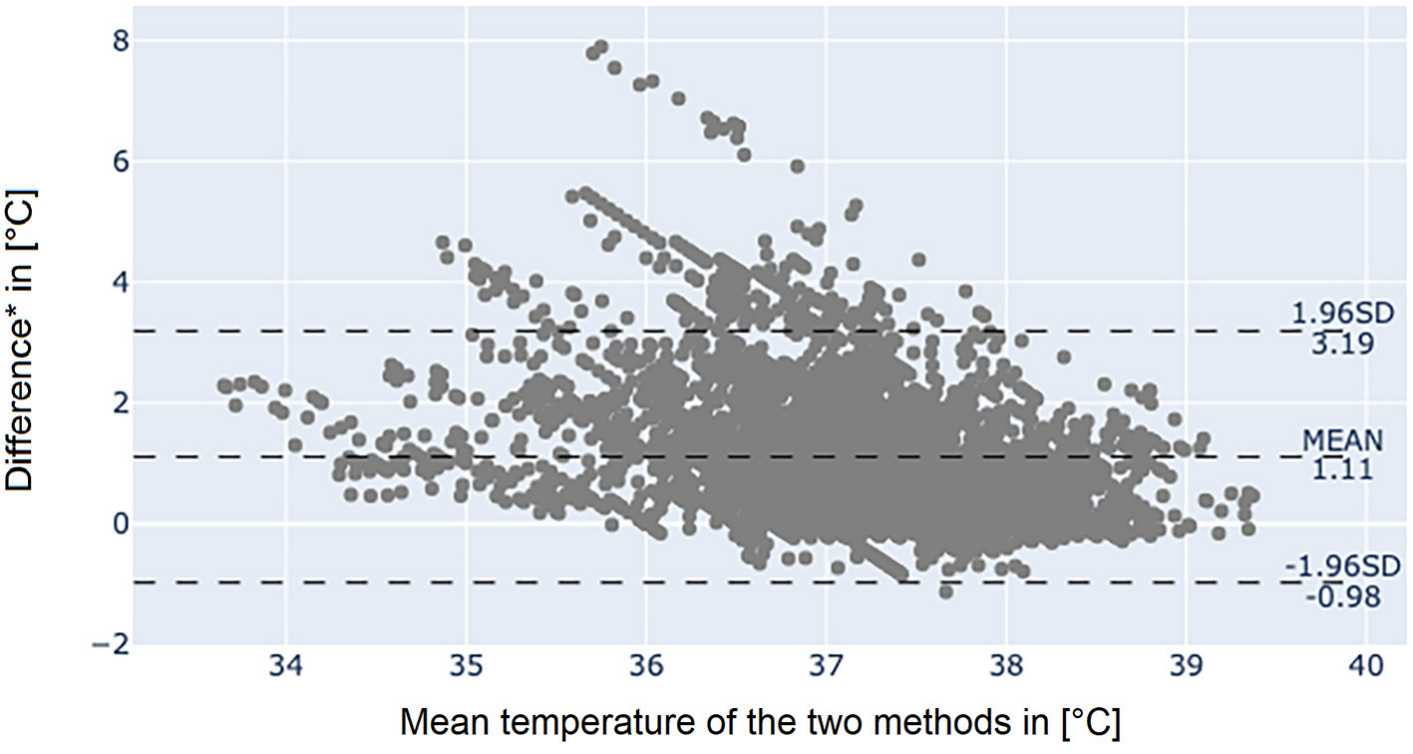
Bland-Altman plot between bladder catheter and the adhesive axillary thermometer Steady Temp. *Bladder catheter values minus adhesive axillary thermometer values. SD, standard deviation.

The Bland-Altman plot of the axillary temperature measurements vs. measurements of the adhesive axillary thermometer as illustrated in Figure 3 showed, that the mean difference (MD) between the conventional axillary method and the new adhesive axillary thermometer Steady Temp is 0.15°C. The upper limit of agreement (ULOA) was 1.30°C, while the lower limit of agreement (LLOA) was −0.99°C. A Bland-Altman plot of bladder catheter vs. adhesive axillary thermometer, as shown in Figure 4, revealed a MD of 1.11°C. Upper limit of agreement was 3.19°C and LLOA was −0.98°C.

**Figure 5:**
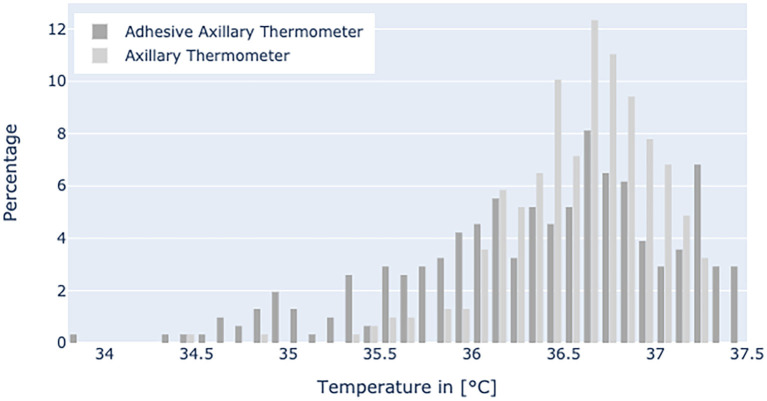
Histogram shows the distribution of the temperature values in the non-febrile group.

Figure 5 displays the temperature range in the non-febrile group with mean values of 36.54°C for axillary thermometer and 36.35°C for patch temperature values, respectively.

In order to calculate sensitivity and specificity we defined a value of ≥38°C assessed with bladder catheter as fever. Cut-off for axillary and patch values was ≥37.2°C for the definition of fever (10). Data of the healthy subject group, where core temperature was not available, was rated as non-febrile.

Sensitivity for axillary measurement was 0.701 (95% CI: 0.577–0.807) and specificity was 0.950 (95% CI: 0.922–0.970). Positive predictive value (PPV) was 0.723 (95% CI: 0.598–0.827), while negative predictive value (NPV) was 0.945 (95% CI: 0.916–0.966) (*p*-value < 0.0001).

Sensitivity for Steady Temp patch measurement was 0.672 (95% CI: 0.546–0.782) and specificity was 0.866 (95% CI: 0.827–0.900). Positive predictive value was 0.484 (95% CI: 0.379–0.590) and NPV was 0.934 (95% CI: 0.902–0.958) (*p*-value < 0.0001).

**Figure 6:**
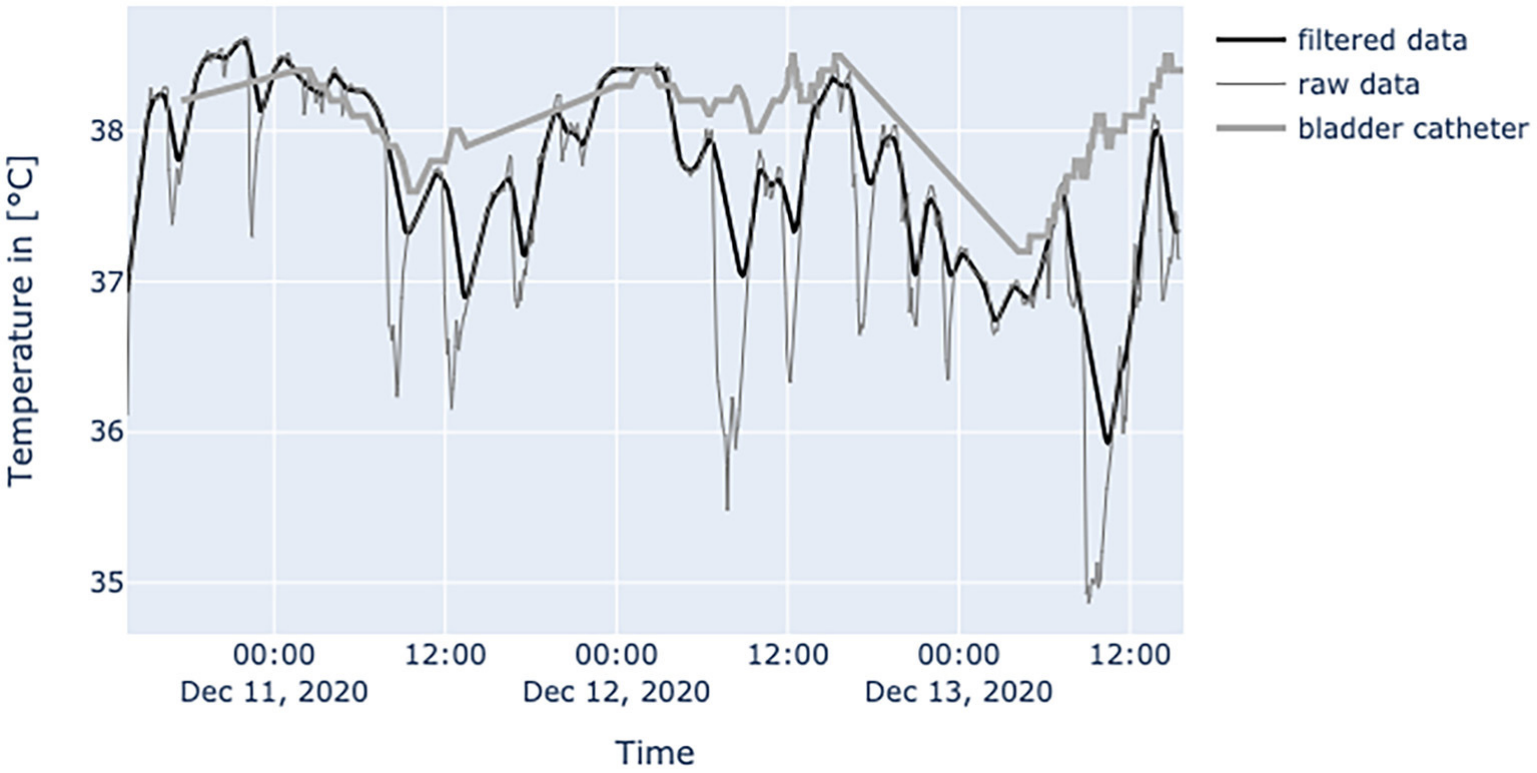
Diagram shows data from a female patient, 79 years old with nosocomial pneumonia. Even with lots of environmental factors distorting peripheral temperature values the trend of core temperature and fever can be estimated sufficiently.

**Figure 7:**
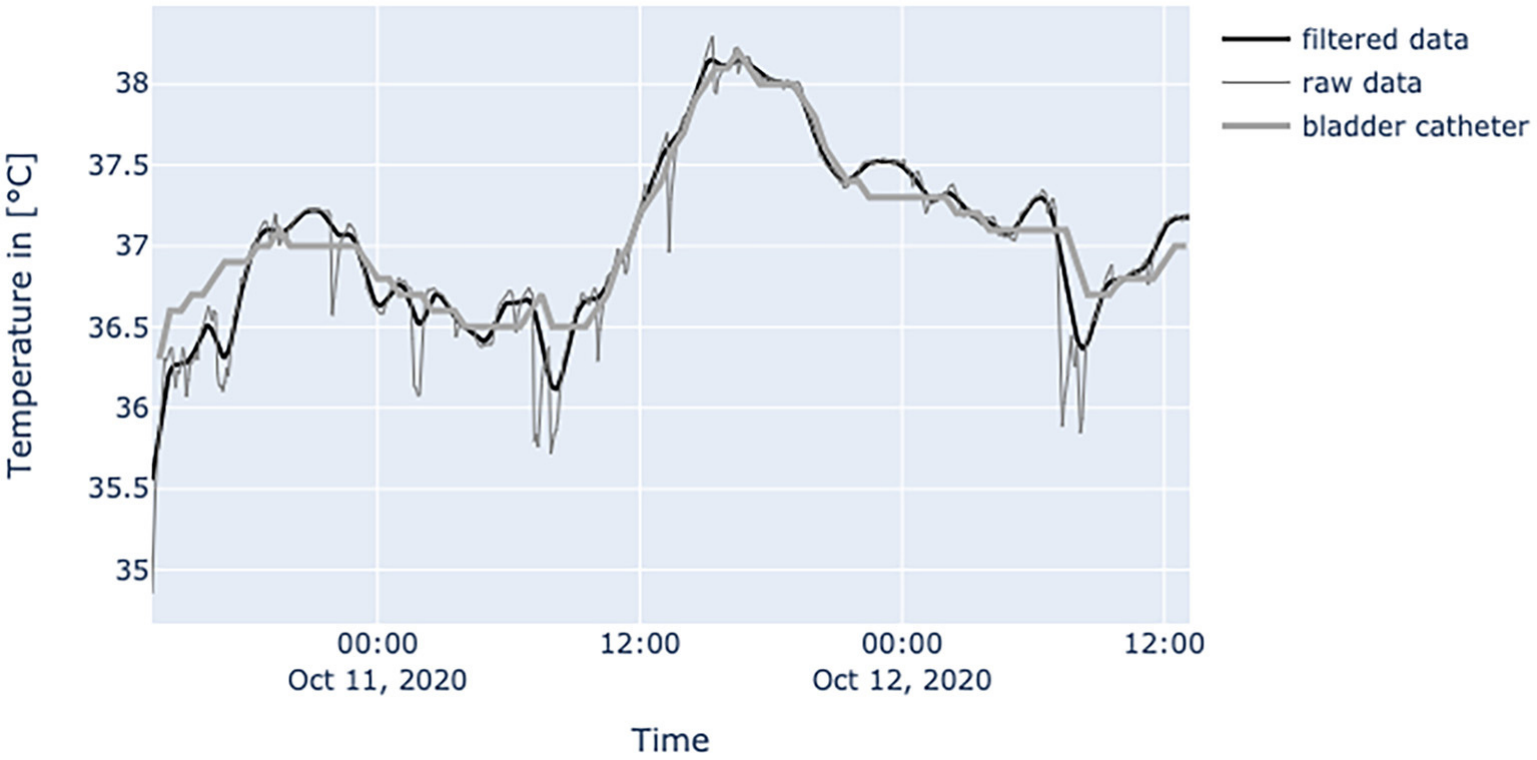
Diagram shows values of a patient with BMI of 24.7 at the age of 71 years, with little movements inside the bed and without the use of catecholamines. Measured temperature is definitely compatible to values obtained by bladder catheter measurement.

**Figure 8:**
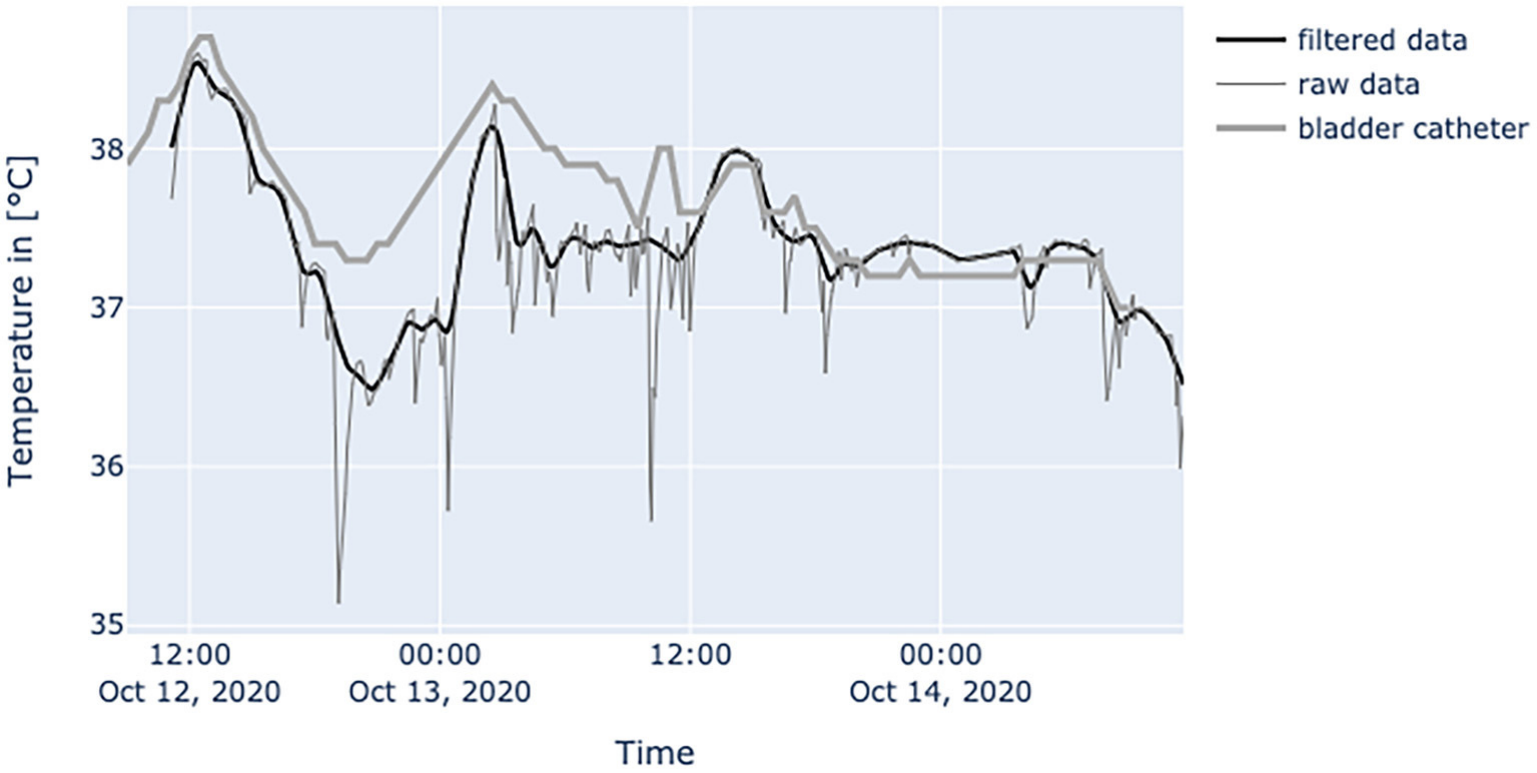
Diagram shows temperature values of a septic patient, who received 1 g acetaminophen and 1 g metamizole on Oct.12, 2020 at 1 p.m. When temperature falls, the effects on the peripheral site are much more severe and can be seen over a prolonged period of time. Raw temperature values nearly reaching 35°C.

**Figure 9:**
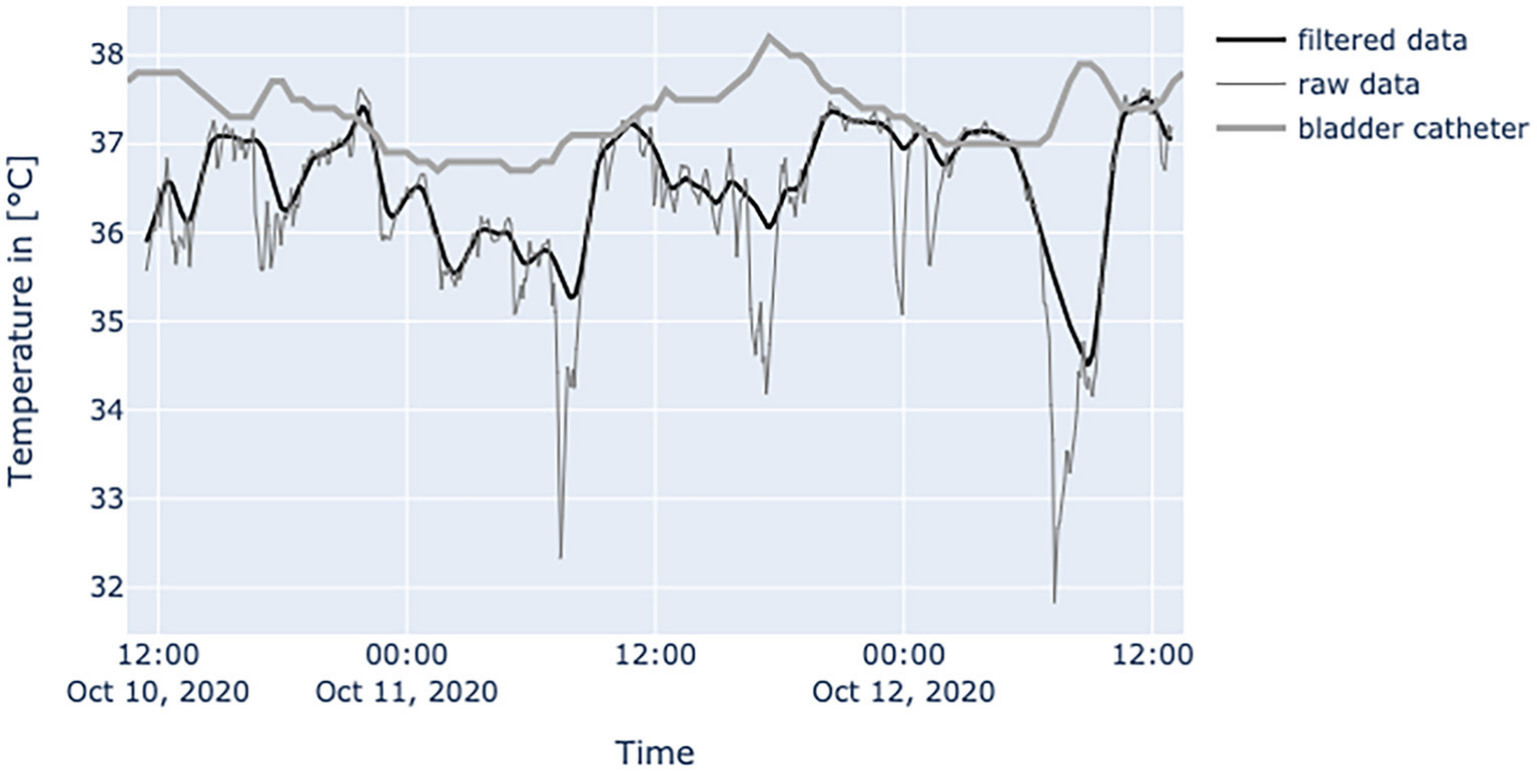
Diagram shows values of an obese (BMI: 34.6 kg/m^2^) patient at the age of 52 years, in relatively good condition, often fully exposing the skin area around the patch to environment. In a patient with this amount of disturbances only the peak patch values should be used. Errors can be easily interpreted by the spikes downwards in raw data.

**Figure 10:**
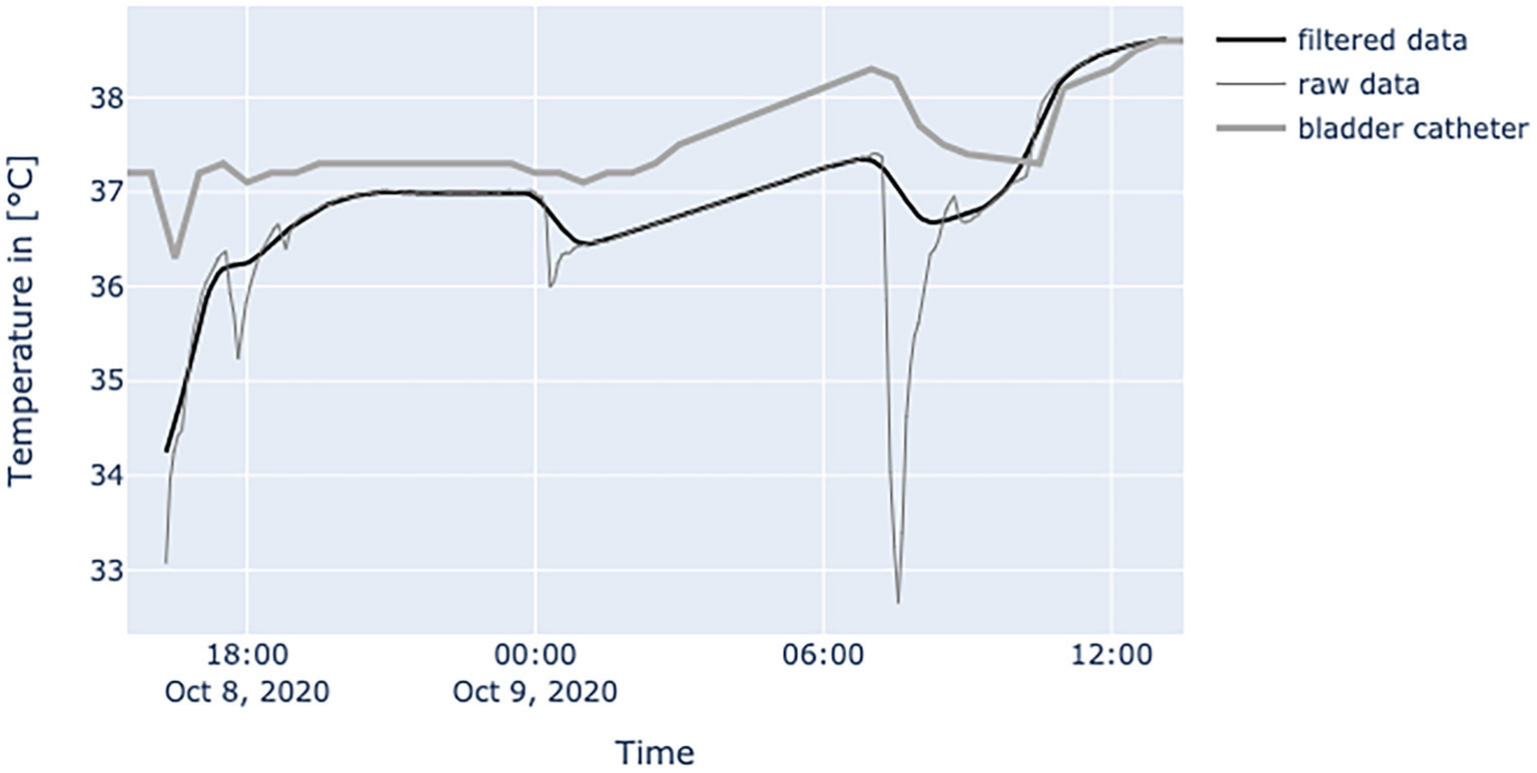
Diagram shows a patient with high vasopressor usage (norepinephrine: 0.401 μg/kgBW/min, terlipressin: 0.02409 μg/kgBW/min) and nearly no movement inside bed. Terlipressin was discontinued at 08:00 and norepinephrine was cut in half at 09:00 (0.2008 μg/kgBW/min). After that intervention, the patch's temperature values adjusted to core temperature.

Figures 6–**10** provide examples of the results of both measurement methods (being bladder catheter and the adhesive axillary thermometer displayed as raw and filtered data).

No skin reaction or any other adverse events during the study period have been identified. The feedback of conscious patients was positive, and they stated that they did not really feel the applied patch.

## Discussion

This was the first clinical study to determine the characteristics of the SteadyTemp® adhesive axillary thermometer. Although the primary endpoint could not be met, the study provides interesting insights in terms of novel possibilities regarding the determination of patient body temperature by using this medical device. Through this novel system, the definition of fever could be performed patient-specifically through relative assessment of the temperature curve of the individual. Blood is the medium of heat transfer in the human body as it has a high thermal capacity (15). In conventional axillary temperature assessment, it is desired to measure the heat transported by the axillary artery (16). It is known that axillary measurements are highly affected by environmental factors such as ambient temperature, local blood flow, underarm sweat, and sufficient/insufficient closure of the axillar cavity during the read-out (6). Since the patch is placed below the axillar cavity, the influence of environmental factors could be amplified due to the measurement location. This results in time periods where the adhesive axillary thermometer produces false low values. However, when the patient is in a resting position, their arms are in contact with the torso and thus, the application area of the patch is covered, which allows accurate continuous measurement of axillary temperature over extended time periods. These measurements can then provide an accurate and reliable estimation of the patient's body temperature, as shown in Figure 6.

The results of the Bland-Altman plot of the SteadyTemp®-system against core temperature measured by bladder catheter showed large differences between the methods (MD of 1.11°C) but might underestimate the clinical agreement. The underestimation can be partially accounted to physiological and environmental influences. However, if a thermal equilibrium was reached, values of the adhesive axillary thermometer agreed well with core temperature. Furthermore, a time shift between the two methods was visible, resulting in less agreement when two values at the same time were compared. This time shift is the result of physiological phenomena. For example, when fever occurs and the set point in the hypothalamus increases, the body tries to store heat, resulting in a peripheral vasoconstriction (17). Hence, an increased difference between the two measurement sites can be observed, until the higher thermal set point is reached, and peripheral perfusion increases again. Another influencing factor is the application of anti-pyretics, leading to mechanisms to release heat, such as sweating (17). Due to the wet skin, sweating results in an evaporative heat loss and a prolonged drop in peripheral temperature values.

Bland-Altman plot showed no noteworthy MD (0.15°C) between axillary measurement and patch values but revealed a variability, which does not allow to interchangeably use these methods to estimate body temperature. It is important to note that our febrile cohort was composed of patients at the ICU, where axillar and peripheral measurement sites are considered unreliable and have a high variability when compared to core temperature (1, 11, 13, 18, 19). Sensitivity of the adhesive axillary thermometer was similar to axillary measurements (0.672 vs. 0.701) and specificity was better for conventional axillary measurements (0.866 vs. 0.950). This may be due to the higher variability of the adhesive axillary thermometer. The higher environmental influence, and the underlying algorithm may affect the ability of the device to produce exact values at a given time.

The benefit of the adhesive axillary thermometer is the detection of changes in the individual's temperature levels. Recent data suggests that there are great difference in normal body temperature inter-individually (4, 5). Furthermore, there are intra-individual changes e.g., due to circadian rhythm [up to 0.9°C daily difference (20)] or menstrual cycle (3). Hence, a single cut-off seems to be too simple to define a possible pathological elevated body temperature. To date, fever definitions in relation to individual temperature levels are only established in a small population, which complicates a general consensus (21).

The interpretation of the generated data is different as obtained values of established measurement methods at the peripheral sites and results from physiological mechanisms and the characteristics of the adhesive axillary thermometer. Axillary measurements are known to have a high specificity for fever, while they lack sensitivity when compared to methods for measuring core temperature (12). Only the core temperature is regulated in a small range of about 1.5°C (2), which is partly achieved by isolating the core via vasoconstriction in peripheral layers to store heat, or vasodilatation to release it (17). Therefore, in a state of peripheral vasoconstriction (e.g., cardiogenic shock), the outer body shell is cooling, creating a gap between core temperature and measured peripheral temperature. Since the patch is placed about 3–5 cm below the armpit, it could be more affected by changes in skin blood flow. When the brachium covers the patch, a great area of skin around the patch is covered too, thereby establishing a thermal equilibrium over time. Hence, the surrounding temperature equals the skin temperature at the measurement site of the patch, resulting in an increase in blood flow and another increase in skin temperature, while heat loss is minimized (22). In patients with little movement, this leads to superficial body temperature values resembling core body temperature over time. This can be seen in Figure 7. In a stable indoor environment, temperature is lower than core temperature. According to thermodynamics heat flow always progresses from core to the outer shell, driven by gradient in temperature. Therefore, the variance in heat loss leads to a difference between peripherally measured temperature values and actual core temperature, which is the variable of interest when measuring body temperature, while other measurement sites only provide a surrogate. Hence, the peripheral measurements tend to be lower than actual core temperature and can never really exceed actual core temperature. With these premises, the values generated by the adhesive axillary thermometer are most reliable at their upper peak of spikes, while creating lower values when environmental disturbances occur. With raw data values of the patch, spontaneous and acute temperature drops can be easily interpreted as environmental disturbances while steadily decreasing temperature values over an extended period of time can be interpreted as a decline of the actual body temperature or decreased peripheral blood flow. Possible errors of the gathered temperature values include strongly sweating patients, and exposure of the measurement site as well as states of hemodynamic centralization. If the patient sweats excessively, the patch becomes moist under the axilla. If exposed to the environment, this can result in a fast drop in temperature values. Because the patch stores the humidity longer than the naked skin, prolonged lower values, as a result of evaporative cooling, can be observed. If the skin area around the patch is not exposed, there is no excessive cooling to be expected. Figure 8 illustrates the effects of sweating on the body temperature recorded by the adhesive axillary thermometer. When the skin around the patch is fully exposed, false low temperature values occur because of the cooling of the skin in this area (15), which is illustrated in Figure 9. In acute illness (e.g., acute heart failure), circulation gets centralized because of an activation of the sympathetic nervous system resulting in vasoconstricted blood vessels of the skin (17). The SteadyTemp®-patch is applied 3–5 cm under the armpit, therefore measuring to a great amount skin temperature different to conventional axillary measurement where heat comes particularly from the axillary artery (16). The effects of vasoconstriction on superficial body temperature are illustrated in Figure 10. This shows that generated data depends to a certain degree on circulation and is susceptible to environmental disturbances, which is common for peripheral sites (6). Due to the continuous measurement with the adhesive axillary thermometer, values with distortions can be recognized and the interpretation can be performed on values with a low degree of distortion.

The possibility of continuous readings of body temperature and the relative changes of body temperature in the individual allows a broad spectrum of different application fields. Normal range of body temperature is known to be highly variable inter-individually (4, 5). Hence, it might be necessary to establish new definitions of fever based on the individual (3), as already established for the elderly (>65 years), where fever is also defined as an increase of >1.1°C over base line temperature (21). Older people are observed to have a lower temperature (21, 23, 24), often not reaching the absolute cut-off values generally accepted as fever. On the other hand, this population is at serious risk to develop infections (21), one major cause of fever. In addition, older people have attenuated mechanisms to adapt skin blood flow in thermal stress (25). While this is generally not favorable, here it could lead to better agreement between peripheral and core temperature values as core temperature changes will project better at the peripheral site. In this population, the continuous measurement and relative interpretation of temperature values the adhesive axillary thermometer provides, could be additionally beneficial. Also immunocompromised patients often cannot mount fever due to their underlying condition (1). Therefore, similar to older people, the adhesive axillary thermometer can improve the evaluation of their individual health condition. In long-term care facilities or hospitals, the SteadyTemp®-system could be a surveillance tool for patients at risk to develop an infection. Once the patient is registered in the app, additional readouts will be assigned to this patient. Hence, mistakes in the assignment can be ruled out. Stations can be implemented to let the patient read out their own patch, sending data immediately to the digital patient record, optimizing workflow. Individual base line temperature levels can be documented to refer to at future visits. Another application lies in recognizing fever patterns. Characteristics of them can often give important hints on the underlying cause and lead to the next diagnostic steps, verifying the diagnosis (17). Furthermore, the circadian rhythm can be monitored in certain disorders such as depression (26).

In acute settings, e.g., acute heart failure, the adhesive axillary thermometer should not be used. Overstimulation of the sympathetic nervous system will often lead to peripheral vasoconstriction, e.g., cutting off the transport of heat to the skin, while additionally excessive sweating is triggered (27), both conditions resulting in an underestimation of the peripheral temperature values. Table 2 summarizes the benefits as well as shortcomings of the adhesive axillary thermometer, the SteadyTemp®-system.

**Table 2:** Summary of advantages and disadvantages of the adhesive axillary thermometer.

**Pros**	**Cons**
Continuous measurements	Susceptible to environmental disturbances (e.g., continuous exposure, excessive sweating)
High comfort	Not suitable for acute circumstances
Easy to use	More expensive than established non-invasive methods
Can detect the individual temperature level	

## Conclusion

This is the first clinical evaluation of the SteadyTemp®-system indicating that it can be a useful device for surveillance, especially in populations which often do not reach absolute temperature levels recognized as fever by point wise peripheral measurement. Also, it could be a diagnostic tool in characterizing fever patterns.

As the adhesive axillary thermometer lacks the ability to measure exact values at a given time and interpretation of data relies to a certain degree on longer observation periods, it should not be used in acute settings e.g., emergency room.

This study is another indicator that the arbitrarily established definition of fever is outdated and based mostly on tradition rather than on recent data. With the ability to easily perform continuous non-invasive temperature measurements, a definition based on the individual's base line temperature seems accurate and practical. As a next step further studies are needed to establish new cut-offs for certain diseases (e.g., infectious diseases) for this particular device based on the relative temperature profile of the individual. Furthermore, studies are needed to verify an improved outcome using this new device as well as to demonstrate the effectiveness in optimizing workflow in the clinical routine.

## Data Availability Statement

The raw data supporting the conclusions of this article will be made available by the authors, without undue reservation.

## Ethics Statement

The studies involving human participants were reviewed and approved by ethical committee of the Medical University of Graz, Austria. The patients/participants provided their written informed consent to participate in this study.

## Author Contributions

JE, MF, KS, WK, and RK contributed to conception and design of the study. JE organized the database. JB performed the statistical analysis and wrote the first draft of the manuscript. JB, JE, KS, and RK managed the febrile group in the study. MS and GW managed the non-febrile group for the study. All authors contributed to manuscript revision, read, and approved the submitted version.

## Conflict of Interest

The study was financed by the SteadySense GmbH, Seiersberg-Pirka, Austria. JE, KS, MF, and WK is employed by SteadySense GmbH. This study received funding from SteadySense GmbH. The funder had the following involvement with the study: data collection, data analysis, study design, preparation of the manuscript and decision to publish. MS and GW is employed by Das Kinderwunsch Institut Schenk GmbH. The remaining authors declare that the research was conducted in the absence of any commercial or financial relationships that could be construed as a potential conflict of interest.

## Publisher's Note

All claims expressed in this article are solely those of the authors and do not necessarily represent those of their affiliated organizations, or those of the publisher, the editors and the reviewers. Any product that may be evaluated in this article, or claim that may be made by its manufacturer, is not guaranteed or endorsed by the publisher.
